# Supplementary material for: From the cage to the wild: introductions of Psittaciformes to Puerto Rico
Source: PeerJ. 2018 Oct 30;6:e5669. doi: 10.7717/peerj.5669 (PMC6214232; doi:10.7717/peerj.5669)

S5: Distribution of Psittaciformes in Puerto Rico based on location records from *Ebird* (1960–2017).

Peach-faced lovebird
(*Agapornis roseicollis*)

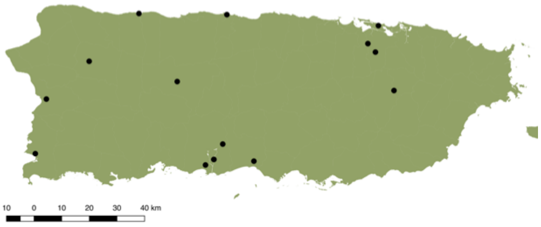

Blue-fronted amazon
(*Amazona aestiva*)

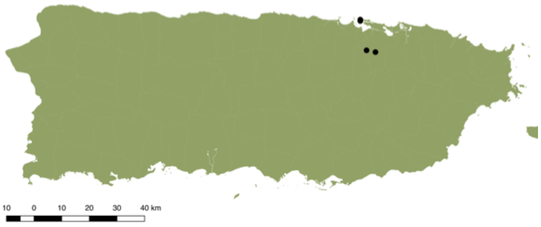

White-fronted amazon
(*Amazona albifrons*)

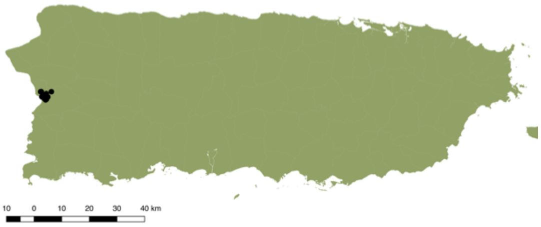

Orange-winged amazon
(*Amazona amazonica*)

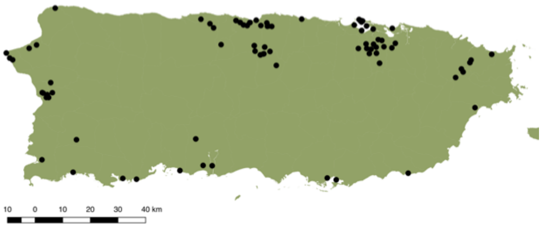

Yellow-headed amazon
(*Amazona oratrix*)

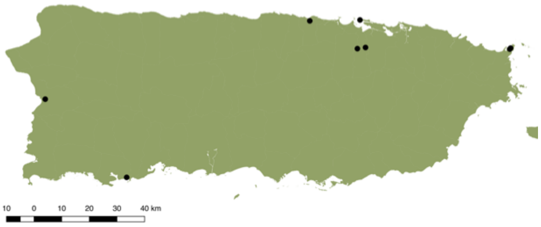

Hispaniolan amazon
(*Amazona ventralis*)

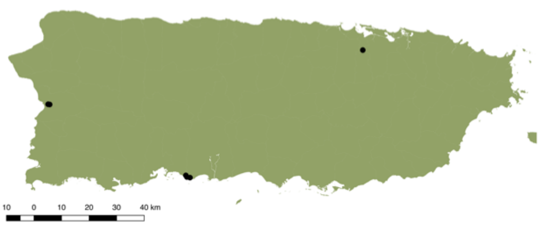

Greencheeked amazon
(*Amazona viridigenalis*)

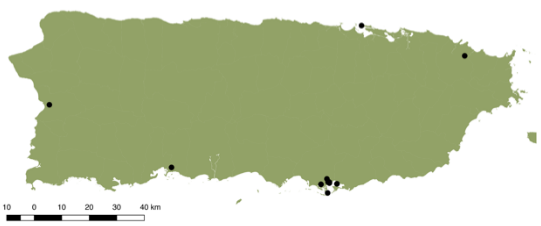

Blue-and-yellow macaw
(*Ara aranaura*)

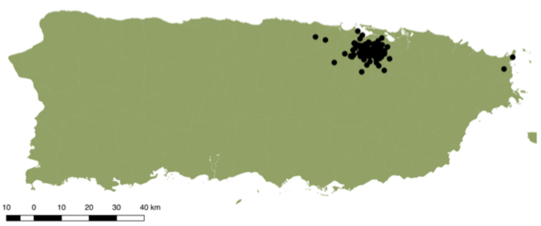

Red-and-green macaw
(*Ara chloropterus*)

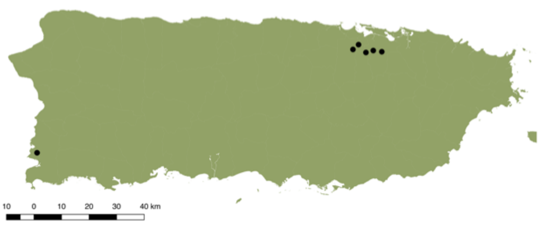

Scarlet macaw
(*Ara macao*)

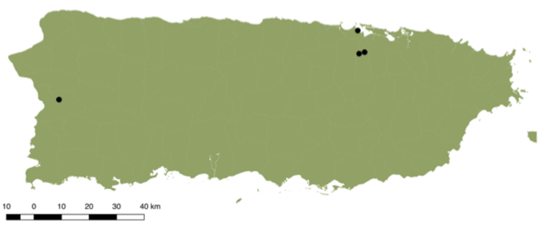

Military macaw
(*Ara militaris*)

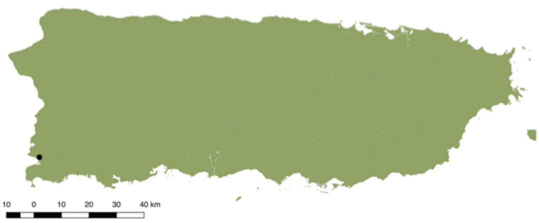

Sun conure
(*Aratinga solstitialis*)

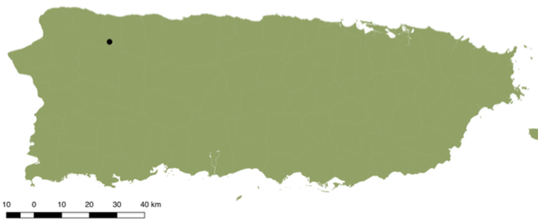

White-crested cockatoo
(*Cacatua alba*)

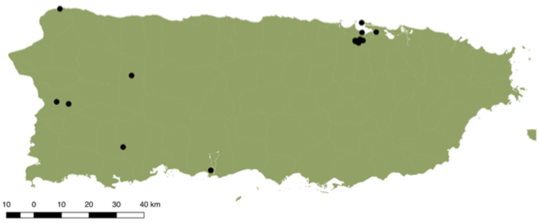

Sulfur-crested cockatoo
(*Cacatua galerita*)

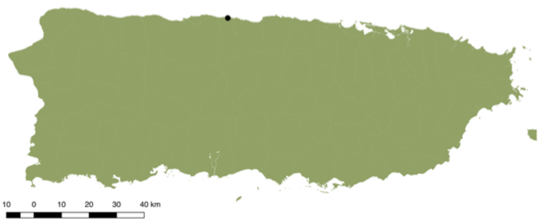

Goffin's corella
(*Cacatua goffiniana*)

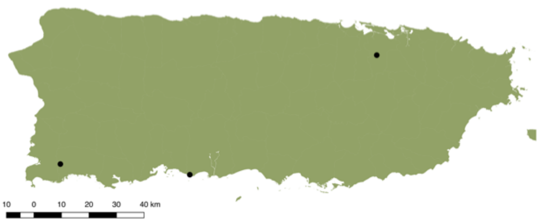

Salmon-crested cockatoo
(*Cacatua moluccensis*)

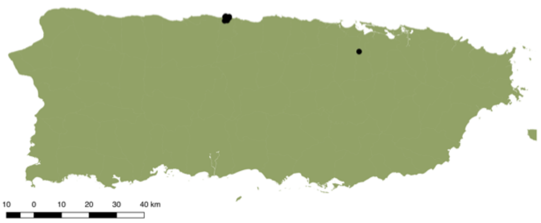

Brown-throated conure
(*Eupsittula* (*Aratinga*) *pertinax*)

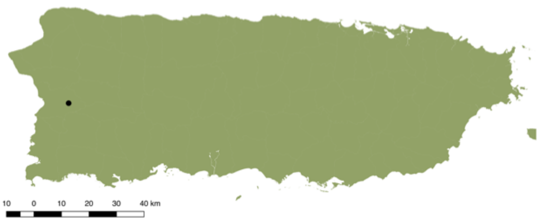

Budgerigar
(*Melopsittacus undulatus*)

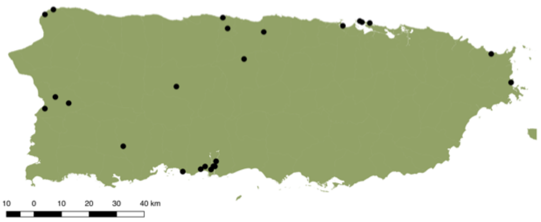

Nanday conure
(*Nendayus* (*Aratinga*) *nenday*)

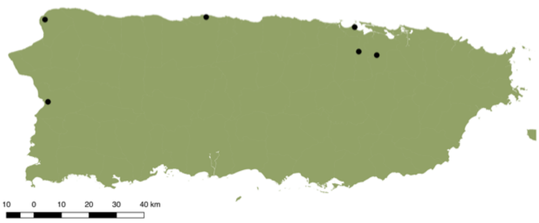

Cockatiel
(*Nymphicus hollandicus*)

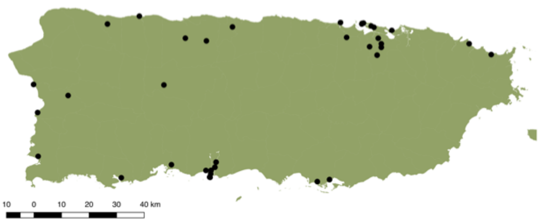

Hispaniolan conure
(*Psittacara* (*Aratinga*) *chloroptera*)

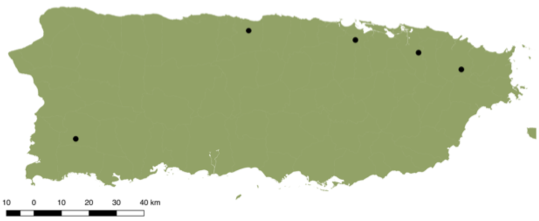

Roseringed parakeet
(*Psittacula krameri*)

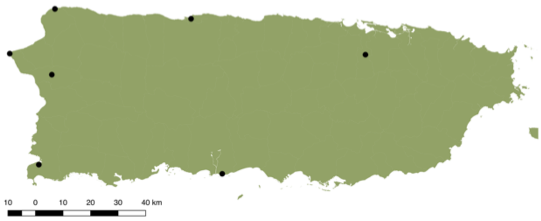

Green-cheeked conure
(*Pyrrhura molinae*)

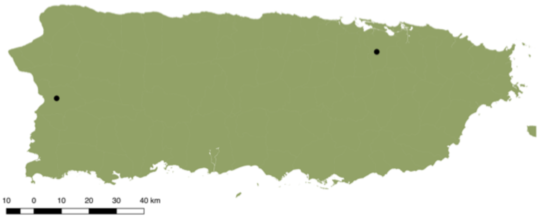

Supplement: Supplemental Information 1 — S1: Puerto Rico psittacine species checklist and status S2: Psittacine species occurrence locations and counts S3: Puerto Rico Landcover classification scheme S4: Psittacine species sighted by local groups (LGs) in Puerto Rico S5: Distribution of Psittaciformes in Puerto Rico based on location records from Ebird (1960–2017) [file peerj-06-5669-s001.zip › S5 Psittacine species distribution PR_submitted.pdf]
